# Constructing therapeutic support and negotiating competing agendas: A discourse analysis of vocational advice provided to individuals who are absent from work due to ill-health

**DOI:** 10.1177/13634593221148446

**Published:** 2023-04-24

**Authors:** Benjamin Saunders, Carolyn Chew-Graham, Gail Sowden, Kendra Cooke, Karen Walker-Bone, Ira Madan, Vaughan Parsons, Cathy H Linaker, Gwenllian Wynne-Jones

**Affiliations:** Keele University, UK; University of Monash, Australia; University of Southampton, UK; King’s College London, UK; Guy’s and St Thomas’ NHS Foundation Trust, UK; University of Southampton, UK; University of Southampton, UK; Keele University, UK

**Keywords:** theme-oriented discourse analysis, therapeutic support, work absence

## Abstract

Work participation is known to benefit people’s overall health and wellbeing, but accessing vocational support during periods of sickness absence to facilitate return-to-work can be challenging for many people. In this study, we explored how vocational advice was delivered by trained vocational support workers (VSWs) to people who had been signed-off from work by their General Practitioner (GP), as part of a feasibility study testing a vocational advice intervention. We investigated the discursive and interactional strategies employed by VSWs and people absent from work, to pursue their joint and respective goals. Theme-oriented discourse analysis was carried out on eight VSW consultations. These consultations were shown to be complex interactions, during which VSWs utilised a range of strategies to provide therapeutic support in discussions about work. These included; signalling empathy with the person’s perspective; positively evaluating their personal qualities and prior actions; reflecting individuals’ views back to them to show they had been heard and understood; fostering a collaborative approach to action-planning; and attempting to reassure individuals about their return-to-work concerns. Some individuals were reluctant to engage in return-to-work planning, resulting in back-and-forth interactional negotiations between theirs and the VSW’s individual goals and agendas. This led to VSWs putting in considerable interactional ‘work’ to subtly shift the discussion towards return-to-work planning. The discursive strategies we have identified have implications for training health professionals to facilitate work-orientated conversations with their patients, and will also inform training provided to VSWs ahead of a randomised controlled trial.

## Background

Sickness absence from work is a socioeconomic global burden (Silva-Junior and Fischer, 2014). Work participation is known to be beneficial to people’s overall health and wellbeing (Waddell and Burton, 2006), but many people who are absent from work face difficulties in building self-efficacy to overcome obstacles to returning-to-work (RTW) (Aylward, 2016). Intervening early during a period of sickness absence is therefore encouraged to help prevent negative health, social and economic consequences that result from longer-term absence and work loss (Waddell and Burton, 2006). A key difficulty is that accessing vocational advice and support is challenging for many people. For example, in the UK, only around a third of employees have access to occupational health services (Fit for Work Europe, 2020), which leaves the majority seeking occupational support from other health professionals such as General Practitioners (GPs). There are challenges for healthcare professionals in meeting this need, with training and education in managing health and work being paramount (Letrilliart and Barrau, 2012).

Providing early vocational advice and support has been found to be effective in helping people with musculoskeletal pain to RTW sooner. The Study of Work And Pain (SWAP) randomised controlled trial showed that introducing a brief vocational advice service in general practice, delivered by trained individuals, led to an average reduction in work absence of 5 days per employed patient over 4 months with a Return-on-Investment of £49 per £1 invested (Wynne-Jones et al., 2019). The Work And Vocational advicE (WAVE) research programme is building on the success of the SWAP trial, through testing an amended vocational advice intervention, delivered to a broader range of patients presenting in primary care. A feasibility study was conducted in which three vocational support workers (VSWs), from occupational support and counselling backgrounds, delivered vocational advice to 19 people who had been signed-off from work by their GP through receipt of a ‘fit note’. In the UK, fit notes are issued to provide evidence of the advice given by the GP about the individual’s fitness for work. Participants were recruited from six practices in three areas of England: West Midlands, South London and Hampshire.

The WAVE intervention was adapted from the SWAP trial intervention (Sowden et al., 2019), which was based on the Flags framework (Kendall et al., 2009). This framework was developed for use by health and employment professionals, to guide them in identifying and addressing obstacles that people may face in returning to work following a period of sickness. Obstacles are categorised under different coloured flags. Yellow flags indicate psychological obstacles, for example, beliefs about pain, illness behaviours; blue flags indicate work related or social obstacles, for example, beliefs about the physical and social impact of work on health and black flags are systems obstacles, for example, working conditions and characteristics, or the financial impact of working status such as job security and benefit entitlements. The WAVE intervention supported VSWs to work with participants to identify obstacles to working and to use these obstacles to structure the consultation. The consultation also included the development of a RTW plan, tailored to the specific obstacles to RTW that each participant faced.

Uncertainties remain about how vocational advice and support can be effectively delivered in consultations between VSWs and individuals who are signed-off from work. In this article, we report the findings from a study in which we aimed to explore how vocational advice was delivered and received in consultations, investigating the discursive and interactional strategies employed by VSWs and participants to pursue their joint and respective goals.

## Methods

In the WAVE feasibility study, the three VSWs audio-recorded a total of 35 consultations to enable the assessment of fidelity of delivering the intervention, using a fidelity checklist, and for use in the qualitative study. Participants provided written informed consent for their consultations to be recorded and used for analysis, and VSWs reaffirmed consent verbally at the beginning of each recording. The study received ethical approval from the NHS West of Scotland Research Ethics Service, 23.09.20, ref: 20/WS/0127.

**Figure 1.**
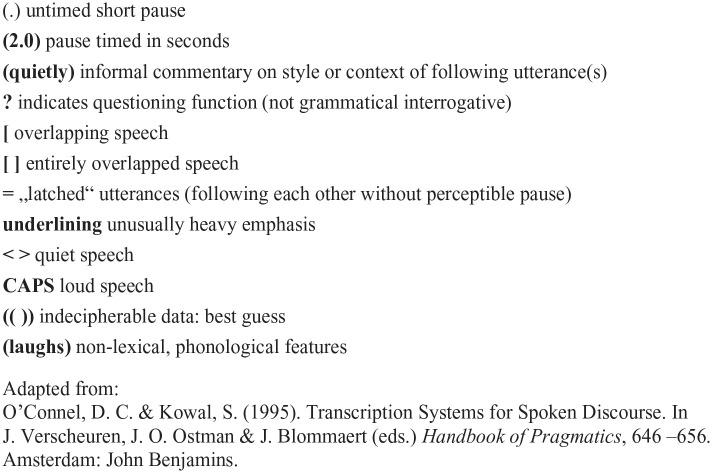
Transcription conventions.

A random sample of eight recordings were selected for analysis. Consultation recordings were initially transcribed for content only, and transcripts were pseudo-anonymised through replacing names, places and any other potentially identifiable information. A second stage of transcription was then carried out, through listening closely to the recordings and adding prosodic and paralinguistic features, for example, hesitations, stress and intonation, as well as timed pauses, interruptions and overlapping speech. The transcription conventions used are displayed in Figure 1.

Transcripts were analysed using theme-oriented discourse analysis (DA) (Roberts and Sarangi, 2005), which involved close examination of VSWs’ and participants’ use of language, focusing on the linguistic features and strategies used to achieve their interactional goals. In theme-oriented DA, ‘themes’ do not refer to substantive data-driven themes, but ‘analytic themes’, which are drawn upon to interrogate the data and draw inferences. The first analytic theme is ‘Face and Facework’ Goffman (1955), and derived from this, Brown and Levinson’s (1987) work on Politeness Theory. Goffman (1955) defined ‘face’ as the ‘positive social value a person effectively claims for [themselves]. . .during a particular contact’ (p. 213); and he proposed that we seek to manage the impression of ourselves in everyday life in order to protect our ‘face’. Brown and Levinson (1987) expanded upon this, positing that ‘face’ has two components that everybody presents, at different times and in different contexts: a positive ‘face’, that is, a person’s desire to be viewed by others in a positive manner, for example, to be liked or admired; and a negative ‘face’, that is, the person’s wish for their actions to be unimpeded by others, for their rights and privacy to be respected. Brown and Levinson argued that in everyday interactions people often attempt to protect their positive and/or negative face, as well as protecting the face of others. This can be achieved through ‘politeness strategies’, which ‘determine how direct or indirect to be, and how far to claim relative closeness and informality or relative distance and formality’ (Roberts and Sarangi, 2005: 39). ‘Protecting’ or ‘Saving face’ also involves avoiding potential ‘face threating acts’, which are actions that may cause somebody to lose face, or damage it in some way. This might include, for example, disagreeing with or contradicting the other person.

A second analytic theme relates to how individuals foreground different aspects of their ‘social and professional identity’ at different times within an interaction, to achieve particular interactional goals (O’Keefe and Shepherd, 1987), such as to strengthen a claim or to build rapport. Thirdly, ‘rhetorical devices’ are strategies individuals use in order to persuade somebody to agree with a particular version of events; including through: the organisation of talk around contrasts, metaphor, analogy and reported speech.

These analytic themes were drawn upon to inform the analysis of VSW-participant consultations. An idiographic focus was initially adopted, with each transcript analysed individually on a line-by-line basis by BS, a researcher from a social science background with experience in using DA. This analysis explored how the interaction developed throughout the course of each consultation, including through use of the three analytic themes. This allowed for the development of a detailed understanding of how the components of the vocational advice intervention were delivered, and how participants engaged with these components. A sample of five transcripts was independently analysed by CCG, a general practitioner (GP) and experienced qualitative researcher. The purpose of this independent analysis was to understand cross-disciplinary perspectives on the data and, through discussion, to come to an agreement on shared meanings and interpretations.

In what follows, we briefly outline details of the consultations and characteristics of the participants, followed by an analysis of extended extracts from the consultations. Whilst all eight consultations were analysed, for reasons of space, the focus here is on extracts from four consultations, chosen as they reflect the discursive patterns frequently observed across the rest of the dataset.

## Findings

### Sample characteristics

The eight consultations analysed included consultations with all three WAVE feasibility study VSWs, involving six patients in total. Consultations were between 21 minutes and 1 hour 19 minutes in length. Six were initial appointments, and two were follow-up consultations. Of the six participants, five were female and one male, aged between late 20s and late 70s. Three participants were absent from work due to a musculoskeletal pain problem and three as a result of anxiety and/or depression.

### Providing therapeutic support and negotiating return-to-work planning

Two main threads running through the data were identified: the ways in which VSWs constructed therapeutic support within consultations; and how VSWs and participants negotiated their goals and agendas for the consultation, which were sometimes shared, but at times were competing.

A key goal for the VSWs was to identify the challenges and barriers participants faced to RTW, and to explore with them how these could be overcome. The participant (P1) in the extract below, had been on sick leave from her job in a school for several weeks due to anxiety and depression. P1 had highlighted to the VSW earlier in the consultation that one of the challenges she faced was communicating with a senior colleague at the school in which she works, and P1 expressed concerns about an upcoming meeting between the pair:

Extract 1

 1  P1: she’s quite intimidating (.)

 2  VSW 1: okay that’s how you experience her yeah (.)

 3  P1: yeah and she like she’s a very (1.0) I mean she’s [senior staff] she has to be (laughs) 4   (.) very like straightforward straight to the point (.)

 5  VSW 1: okay=

 6  P1:   =erm and I do find sometimes (.) it’s (.) hard to explain how I’m feeling (.)

 7  VSW 1: okay (.) so again

 8  P1:     [so I don’t

 9  VSW 1:        [I feel like I’m (.) sort of jumping to potential plans (.) but I’m I’m10   eager to sort of (.) sort of pause when you’re flagging up (.) um any plans (.) that’s 11   part of my role is to is to hold those up to you and say

12  P1:                     [yeah]

13  VSW 1: oh oh potentially (.) you know at some point we could perhaps look at (.) perhaps 14 prior to your meeting with [senior colleague] (1.0) erm (.) what it is that you really 15 want to be saying and and to be heard (.) you know

16  P1:                   [yeah]

17  VSW 1: just to think about you know (.) how to communicate (.) what’s really

18  P1:                           [yeah]

19  VSW 1: I can really hear (.) um how important that is to be heard that you are trying your 20   very best

VSW 1 initially provides support for P1 through validating her feelings of ‘intimidati[on]’ (line 1) towards her colleague, but does so in a way that avoids providing a personal evaluation, instead reflecting P1’s feelings back to her (2). When P1 proceeds to explain in more detail the difficulties she feels in communicating with her colleague, VSW 1 interrupts this explanation (9). VSW 1 provides a justification for this interruption based on their ‘eager(ness). . .to pause’, because they feel P1 is ‘flagging up. . .plans’ (10). This interruption allows the VSW to shift the agenda away from focusing on P1’s description of her colleague, to explicitly discussing making future plans based on the difficulties she described. VSW 1 acknowledges the abruptness of this shift by highlighting that they are ‘jumping’ to potential plans (9), rather than allowing these plans to emerge more naturally. VSW 1 provides further justification for this interruption through framing the identification of these plans as part of their professional role in the consultation (10–11). This shows VSW 1 going ‘on-record with redress’ (Brown and Levinson, 1987), which means that whilst they are potentially threatening P1’s negative face (i.e. her desire not to be imposed upon) in a direct manner through cutting her off mid-utterance, the justification offered is used as a strategy to mitigate this potential ‘face threat’. Through indicating that their role is not to initiate future plans, but to highlight plans indicated by P1 and present them back to her, that is, ‘hold those up to you’ (11), the VSW constructs this as a form of collaborative action planning, with their role being a facilitator of these plans.

In lines 13–15, VSW 1 proposes a potential plan based on the challenges P1 has highlighted; however, rather than suggesting a direct action for P1 to undertake alone, the VSW uses the plural pronoun ‘we’ to suggest collaborative action in the form of a future discussion between them, to develop a plan for the upcoming meeting with the senior colleague. Through this suggestion – the force of which is mitigated through the modal verb ‘could’ and repetition of ‘perhaps’ (13) – the VSW is able to introduce these plans as tentative and as-yet-undecided. This appears to show recognition of P1’s reluctance to begin RTW planning that was expressed throughout the consultation, and therefore reflects an attempt to encourage her to engage with RTW planning through an indirect, gentle approach. This indirect approach appears to mitigate the face threat that may arise from VSW 1 challenging P1’s reluctance to engage in RTW planning. In doing so, VSW 1 is able to protect P1’s ‘negative face’ (Brown and Levinson, 1987).

VSW 1 also displays empathy and solidarity through explicitly articulating that they have listened to, and understood, P1: ‘I can really hear’ (19). They validate P1’s proposition from earlier in the consultation that she is ‘trying [her] very best’ (19–20) to self-manage her anxiety and depression, and her concern that this has not been acknowledged by her workplace. The prosodic stress on ‘trying’ and ‘best’ further strengthens this validation. This validation functions to support P1’s ‘positive face’, through helping her to sustain the impression of herself as somebody who is conscientious, and is putting in the work to overcome the challenges she faces.

The following extract shows VSW 2 adopting a similarly supportive approach to engage in subtle negotiation of RTW discussions. The participant (P2) had been absent from her job working with young people for several months due to stress and anxiety, as well as persistent upper-limb pain. Up to this point in the consultation P2 had expressed reluctance to engage in conversations about RTW planning:

Extract 2

21  VSW 2: how do you feel (.) about (.) returning to work overall? What are your22 thoughts? (.) So I know it might not be (.) erm (.) something that’s going to23 be happening imminently (.) but

24  P2:            [mmm]

25  VSW 2: overall what are your thoughts? (1.0)

26  P2: erm (1.0) it’s been my career (.) so I’m you know like I say with forty years’27 service since I was sixteen (.) erm (1.0) I enjoy my work with my young28 people (.) you know (.) setting plans for them what they’re going to do and29 it’s a nice job in the respect of (.) I can work with a young person from when30 they’re eighteen right the way through till they’re (.) you know twenty one31 definitely (.) erm but I’ve got some that are twenty two twenty three=

32  VSW 2:                            =yeah=

33  P2:                               =and 34 to see that transition from them you know (.) what what they do achieve (.)35 in that (.) and you know I’ve got some lovely young people that (.) you know36 (.) are are important and their lives are all important but you know just doing37 that (.) that work (.) to set them off to independence really (1.0) I love the38 job and I don’t want to give up my job (1.0) I just need it to be manageable39 and not go through what the last year’s been (.)

40  VSW 2: No no I completely understand but that does that’s (.) really promising (.) the41 way you’ve described it (.) so it’s job satisfaction you can’t get anywhere else42 is that you won’t get that in another job (.) erm (.)

43  P2: yeah=

44  VSW 2:   =or similar like that (.) erm (1.0) the fact that you love your job and45 enjoy it (1.0) are satisfied by it is probably one of the most important things46 because that’s going to be a drive to help you get back to it isn’t it? (.) Erm

47  P2:                               [Yeah 48 (.) yeah (.)

49  VSW 2: it’s just about getting that (.) getting it managed (.) the caseloads (.) the50 workloads (.) the the stress levels (.) be listened to a little bit more when51 you’re having issues

VSW 2 uses an open question to explore P2’s views and feelings about RTW (21), but qualifies this with the assurance that they are not referring to this happening ‘imminently’ (23), thereby acknowledging P2’s earlier assertions that she did not yet feel ready to consider returning. Rather than directly responding, P2 provides a lengthy explanation of her enjoyment of her job (26–38), emphasising the meaningful role work has played in her life through highlighting her long service (26–27). P2 summarises that, ‘I love the job’ (37–38), but then expresses the concern about losing her job, which she speculates could happen if she were to ‘go through what the last year’s been’ (39), implicitly referring to the stress she experienced prior to her sickness absence, as described throughout other parts of the consultation.

VSW 2 explicitly validates these concerns through displaying empathy: ‘I completely understand’ (40). However, they use the conjunction ‘but’ to shift the emphasis away from P2’s concerns and to focus on the positive aspects of her account, evaluating the positivity she expressed for her job as ‘really promising’ (40). Building on this positive evaluation, the VSW subtly moves the conversation back to the issue of RTW, proposing that P2’s ‘love’ of her job can positively influence her behaviour, through providing her with the ‘drive’ (46) to help her resume work. VSW 2’s assertion that this is ‘one of the most important things’ (45) functions to reassure P2, attempting to offset her concerns about having to leave her job role.

The VSW then reflects back to P2 the concerns she had expressed at an earlier stage in the consultation about the perceived lack of support she has received from her workplace. However, VSW 2 reformulates these concerns in a more positive light, mitigating their severity through the phrases ‘it’s just’ (49) and ‘a little bit more’ (50). In this way, VSW 2 attempts to implicitly reassure P2 about her concerns regarding RTW, through positioning her enjoyment of her job and job satisfaction as the principal factors, and her concerns about lack of workplace support as a secondary issue that can be resolved.

The participant (P3) in the following extract also expresses hesitancy to engage in discussions about RTW planning, leading to a back-and-forth negotiation of competing agendas within the consultation. P3 had been absent from her job working with children for a few months due to persistent lower-limb pain:

Extract 3

52  VSW 1: in terms of (.) uh thinking about (.) returning to work (1.0) erm (.) erm53 (.) I’m not sure what your discussions have been with your managers54 about (.) any plans to return to work whether you’ve

55  P3:                    [well]

56  VSW 1: have there been

57  P3:      [no]

58  VSW 1: specific discussions?

59  P3:       [they (.) that the the plan is that (.) the well I’ve had this60 (.) the doctor [name removed] has given me this time over summer (.)61 to get the physio (.)

62  VSW 1: right (.)

63  P3: to try (.) and if that works brilliant then I would go back to work (.)64 although I’ve got a fit note at any point in time (.) if I got up tomorrow65 morning and (.) this was gone (.) I’d be (.) ‘can I come back?’ (.) and66 I’ll work in the play scheme (.)

67  VSW 1: Okay

68  P3:   [but that that’s (.) you know (1.0) and they know that you know (.)69 the

70  VSW 1: [and have they suggested any adjustments in terms of (.) erm (.) yes71 whilst you’re waiting (.) I I absolutely appreciate that yeah waiting til72 treatments start (.) however

73  P3:          [yeah] (.)

74  VSW 1: erm (.) you know an earlier return to work (.) I’m just wondering if75 your managers have (.) um explored with you any plans (.) to you know

76  P3: [well (.) a-a-

77  VSW 1:  [make some adjustments um uh and make it perhaps a little bit easier78 for you? (1.0)

79  P3: [sighs] (1.0) well that would that would have to be (.) me relying on80 other members of staff to do (.) a lot more work=

81  VSW 1:                     =mhm=

82  P3:                        =so where if83 there was three of us working in a day (.) it’d be like two and half84 bodies (.)

85  VSW 1: right (.)

86  P3: wouldn’t it do you know I mean you’ve you’ve you’ve got to work with87 children to (.) it’s a play (.) we’re not we’re not sitting and sitting and88 just doing nice little activities on a table (.) these kids are robust (.)

VSW 1 explicitly introduces the topic of RTW planning (52–58), asking P3 if any plans have been discussed with her managers. The indirect question form (53), following several hesitations, perhaps shows the VSW’s recognition of P3’s earlier resistance to discuss RTW planning. P3 initially begins to respond directly (59) but then shifts the focus of her response, appearing to deflect discussion of RTW on the basis of her healthcare needs taking priority. She implicitly draws on her doctor’s advice to support this stance, citing her doctor having ‘given me this time’ (60) to receive physiotherapy. She provides further justification for this lack of willingness to engage in RTW discussions through suggesting that her RTW is dependent on this treatment improving her condition (60–64). However, whilst she implies that RTW discussions are unsuitable at this point, she also engages in positive ‘facework’ through stressing her desire to RTW if her condition were to resolve (64–66), thereby establishing that it is not her lack of motivation to RTW that is the issue, but her inability to do so. This positive facework appears to function as a face-saving strategy, that is, P3 mitigates any potential threat to her own ‘positive face’ by portraying an image of herself as a dedicated, motivated employee, who wishes to RTW but is unable to due to circumstances outside of her control.

VSW 1 interrupts (70) to implicitly challenge P3’s stance that RTW planning would not be possible until her health condition resolves, through asking if potential adjustments have been explored with her managers ‘whilst. . .waiting’ (71). Through this, the VSW opens up the possibility for RTW discussions in spite of P3 still awaiting treatment. This could be viewed as what Brown and Levinson (1987) term an ‘off-record’ face-threat, whereby the VSW challenges P3’s proposition, but does so through an indirect formulation (74), and the phrase ‘perhaps a little bit’ (77), softening the force of this question in order to mitigate the potential threat to P3’s positive face. VSW 1 also does ‘face-saving’ work through explicitly validating P3’s perspective: ‘I absolutely appreciate. . .’ (71). However, P3 again resists attempts to foreground the agenda of RTW planning, constructing a further justification for her stance based on the anticipated negative consequences to other staff members’ workload if she returned to work but was unable to resume full duties (79–84). As well as functioning as a justification, this shows P3 doing further positive facework, portraying herself as good team member, somebody who cares about the needs of her colleagues. In contrast to the VSW’s mitigated language, P3 expresses greater certainty to strengthen this justification (79–80). She then further justifies her position on the basis of the strenuous nature of her work, contrasting the hypothetical scenario of ‘sitting. . .doing nice little activities’ (87–88) when compared to the reality of the ‘robust’ (88) children that she works with. This further advances her argument that it would not be possible to do this type of work whilst her condition is ongoing.

Following further discussion of the nature of P3’s work duties, VSW 1 attempts to bring the topic of discussion back to RTW planning:

Extract 4

 89  VSW 1: So what I can hear very clearly your concerns about (.) you know the demands of (.) 90 the job that you were doing previously (.)

 91  P3: yeah

 92  VSW 1:  [and when you’ve been in contact with your managers (.) I’m not sure if they have 93 said specifically (.) erm (.) whether anything could be offered or put into place? I’m I 94 am hearing your concerns

 95  P3:           [yeah

 96  VSW 1:             [I’m not sure whether they have (.) erm you know 97 discussed anything specific from their point of view?

 98  P3:            [no (.) not (.) not at all (.) not not

 99  VSW 1:                      [okay]

100  P3: not at all (.) not at all (.) no (.) not now I don’t I’m not saying they wouldn’t (.)

101  VSW 1: no okay

102  P3:   [be open to it of course and I think (.) yeah definitely would (.) but I don’t think103 (.) I I can’t see things working out in in the summer holidays for four weeks in the104 holiday (.) we work four weeks and then (.) we shut down which is holiday time and105 then we obviously we resume (.) the um the normal hours (.) and no maybe (.) maybe106 September (.) when we go back (.) that could (.) if things (.) are (.) obviously a better107 situation (.) um (.) and with working in bubbles then (.) we we possibly could but108 without speaking to them I don’t know (1.0)

109  VSW 1: okay

110  P3:  [I don’t know (1.0)

111  VSW 1: so there’s a possibility to explore (.) just a little bit more

112  P3:                      [yeah]

113  VSW 1: in terms of what might (.) be put in place

114  P3:                 [yeah yeah (.)

115  VSW 1: and yes that might (.) um involve other colleagues and I can hear your awareness of116 you know the impact that might have on your on your colleagues

VSW 1 again validates P3’s views, explicitly emphasising that they have listened to, and understood, this perspective (89–90). Whilst this displays a supportive tone, this acknowledgement also functions to mitigate the potential face threat of VSW 1 subsequently introducing a competing agenda, through again asking whether work adjustments had been discussed (92–93). They do, however, reiterate their earlier validating statement: ‘I am hearing your concerns’ (93–94), to emphasise that in re-introducing this question, they are not seeking to undermine P3’s earlier justification.

P3 eventually directly responds to the question of discussions about workplace adjustments, emphatically stating ‘not at all’ (98), which she repeats several times. However, she proposes that whilst these conversations have not taken place, she believes her workplace ‘definitely would’ (102) be willing to talk about adjustments. P3 then further softens her position; having earlier argued that any RTW would not be appropriate whilst her condition is ongoing, she now suggests that RTW may be possible after the summer when her workplace ‘resume. . .normal hours’ (105). Whilst appearing to be more open to discussing RTW adjustments, she displays cautiousness through hedging and modal verbs: ‘maybe’, ‘could’, ‘possibly’ (105–107), as well as marking further uncertainty: ‘without speaking to them I don’t know’ (108).

VSW 1 picks up on this more receptive tone, summarising P3’s new stance (111–113); however, the VSW also displays caution through describing RTW discussions with managers as a ‘possibility’ (111). P3 signals agreement with the VSW’s summary (112 and 114). Having subtly shifted P3’s stance towards more openness to engaging in RTW planning, VSW 1 again shows explicit acknowledgement and validation of P3’s earlier concerns about the impact of her potential RTW on her colleagues (115–116). This appears to show the VSW attending to P3’s positive face, through acknowledging her caring attitude towards her co-workers. This may also function to consolidate the therapeutic relationship between the two, through reassuring P3 that despite the agreement reached, her earlier concerns have not been overlooked.

The participant (P4) in the final extract, a healthcare worker, had been absent from work for 3 weeks due to lower-limb pain. During the consultation she had discussed RTW obstacles relating to her communication with the occupational health department in her workplace, as well as delays in receiving appointments for tests and treatments, which had resulted in her experiencing a sense of resignation:

Extract 5

117  VSW 3: So (.) I’m kind of aware that (.) you’ve actually been very very proactive (1.0) you118 know you’ve been [trying]

119  P4:          [mm]

120  VSW3: very hard (.) you you took some leave (.) to try and you know after your father (.)121 died and you’ve taken a small leave in January and and you’ve tried to [rest]

122  P4:                              [mm]

123  VSW 3: and (.) you know that you tried to look after yourself as best you can and asked for124 help (.) where possible (.)

125  P4: yeah (.)

126  VSW 3: that you’ve spoken to occupational health (.) that you’ve had your physiotherapist (.)127 you’ve done the exercises they told you (.) um (1.0) and that (.) I can understand why128 at the moment you feel that (.) you’re gonna give up (.) asking (.) um (1.0) but do you129 think you could (.) find (.) some erm (.) some motivation (slight chuckle) and some130 strength somewhere to to push this a bit further with occupational health for your131 own benefit? (1.0)

132  P4: Yeah I will I will try again because (.) ah (.) as I’ve mentioned it’s still ongoing and133 it’s affecting my other joints so (.) I will try to and tell them that my recent134 conversations with the unit (.) but (.) still erm (.) the plans that were made were not135 implemented (.) I can (.) try again (1.0)

VSW 3 pays strong attention to P4’s ‘positive face’ (i.e. desire to be viewed positively by others) (Brown and Levinson, 1987), highlighting the positive steps she has already taken to manage her health (117–124), which the VSW evaluates as ‘very very proactive’ (117). VSW 3 further praises these positive health behaviours in highlighting P4’s active engagement with her occupational health department and physiotherapy treatment (126–127). These positive evaluations may function to empower P4 through giving her the confidence that she had been taking the right action.

However, this recognition of previous proactive behaviour is juxtaposed with VSW 3’s suggestion that P4 currently lacks motivation to engage with these professionals (127–128), representing an ‘off record’, that is, indirect threat to P4’s positive face. They mitigate the potential face threat of this assertion by showing empathy with this position: ‘I can understand’ (127), attempting to protect P4’s positive face by highlighting that there are legitimate and understandable reasons for her currently lacking motivation. VSW 3 then subtly encourages P4 to take further action to re-contact the occupational health department, in spite of her feelings of wanting to ‘give up’ (128). The VSW uses mitigated, hedged language, perhaps displaying a concern about undermining the confidence they have attempted to instil through their previous evaluations. VSW 3 also displays understanding of the difficulty P4 may face in taking this action, asking if she could find ‘some strength somewhere’ (129–130). Furthermore, the VSW attempts to persuade her of the positive outcomes of these actions ‘for your own benefit’ (130–131).

P4 signals agreement with this plan to re-contact occupational health, but repeats the phrase ‘I will try’ (132–133), suggesting she feels that the outcome is not in her hands. She emphasises that previous work adjustments that were agreed ‘were not implemented’ (134–135), appearing to signal a lack of optimism for a positive outcome from this contact.

Following further discussion about the work adjustment plan that P4 had previously agreed with her employer, the conversation returns to plans for P4 to contact occupational health:

Extract 6

136  VSW 3: I think one of the ways is (1.0) is to sort of keep raising that with people so actually137 you know very respectfully and politely (.) but keep going back to occupational138 health and saying ‘actually (.) you know this is the situation (.) you know I just want139 some support (.) so that I am safe at work and so that my patients are safe at work (.)140 and you know (.) what can we do? (.) you know what are my options? (.) how can we141 make this work?’ (.) um (.) and then also I think with (.) with your GP to (.) perhaps142 contact your GP and say (.) you know ‘actually this is still ongoing (.) what are my143 options? (.) what else can you do? (.) you know what other tests are there?’ (.) you144 know I know you mentioned a blood test (.) um (.) you know (.) ‘is it possible for145 those to happen at the moment or do we have to wait a little while because of146 Covid?’ (.) um (1.0) and and to just keep reminding people that you are there (.) and147 that you are still in pain (.) um (.)

148  P4: Yes (.) you know because with the GP (.) it’s like (.) erm (.) they they wanted me to149 wait for that musculoskeletal assessment and (.) nothing else was planned (.)with150 them=

151  VSW 3:   =right (.)

152  P4: erm (.) they even said that if I needed to have an MRI it will be (.) the discretion of153 the musculoskeletal team (1.0)

154  VSW 3: Okay (.) and when are you or were you due to have the musculoskeletal (.) meeting?155 (1.0)

156  P4: Oh (.) that’s the one that they said I’m already booked but (.) erm because of Covid157 they don’t know when (.)

158  VSW 3: yes (.) okay

159  P4:     [I’m on a waiting list something like that (2.0)

160  VSW 3: Okay (1.0) so again it might be worth (.) I mean I don’t know if you can email your161 GP (.) but just to (.) again (.) because I think what we’re saying is (.) essentially (.)162 you know (.) actually ‘I’m still here (.) I’m still in pain (.) please pay attention’

VSW 3 gives explicit advice about how P4 might communicate with her occupational health department and with her GP (136–147). Whilst hedged through ‘I think’ (136) and ‘perhaps’ (141), this advice is constructed in a directive manner, using the first person pronoun ‘I’ to construct hypothetical reported speech, that is, the exact form of words for P4 to use. This is formulated as a series of questions for her to pose to these professionals, thereby advocating a proactive approach where the onus is on P4 to seek the required support.

P4 signals agreement (148), but then implicitly highlights barriers to this proactive approach, through expressing uncertainty about her upcoming appointments and investigations (148–159). VSW 3 responds to this implicit resistance, again in a directive manner, advising P4 to contact her GP to follow up on these appointments, though this is slightly mitigated: ‘it might be worth’ (160). The VSW again uses hypothetical reported speech, but in this case does not suggest the precise wording for this conversation, but frames this as a summary of their overall advice to continue to remind her workplace and healthcare professionals that she is seeking support: ‘I’m still here (.) I’m still in pain (.) please pay attention’ (162).

## Discussion

This is the first study in which an in-depth discursive analysis has been carried out on consultations that focus on supporting people to return-to-work following sickness absence. A range of strategies were identified as being utilised by vocational support workers (VSWs) in providing therapeutic support for participants within consultations. These strategies often focused on supporting the participants’ ‘positive face’ (Brown and Levinson, 1987), that is, helping them to sustain a positive impression of themselves. This included signalling explicit understanding or acknowledgement of participants’ views or concerns (‘I completely understand’); positively evaluating the participant’s personal qualities and prior actions in relation to RTW planning (‘you’ve been very proactive’); reflecting the individual’s views back to them to show they have been heard and understood; fostering a collaborative approach to action planning and attempting to reassure participants about their concerns. The positive ‘facework’ achieved through these strategies appeared to have the goal of building trust and fostering a strong therapeutic relationship, as well as empowering participants and building their confidence to engage in plans about RTW.

In some cases, VSWs were also shown to employ strategies to mitigate potential threats to the participants’ positive or negative face that could undermine their relationship; such as explaining or justifying any interruptions to the participant’s turn, or using indirect ‘off-record’ (Brown and Levinson, 1987) formulations to mitigate the face threat of challenging participants’ viewpoints. This was particularly the case when some participants were reluctant to engage in RTW planning in consultations, leading to VSWs employing discursive strategies to subtly shift the consultation agenda towards addressing RTW obstacles and RTW planning. Back-and-forth negotiations were observed, where VSWs and participants attempted to foreground and justify their individual agendas, which at times revealed differing interactional goals (O’Keefe and Shepherd, 1987). This led VSWs to, at times, put in considerable interactional ‘work’ to subtly encourage participants to engage in RTW planning.

### Comparison with previous literature

Much of the previous qualitative literature exploring how occupational challenges are addressed has focused on consultations with GPs and other primary care professionals. O’Brien et al. (2008) reported that patients often sought advice from their GP about work-related issues, but they perceived GPs did not have sufficient time or knowledge to address these issues adequately. We observed similar concerns in a recent study exploring the potential for First Contact Practitioners (FCPs) to deliver vocational advice to patients absent from work with musculoskeletal pain (Saunders et al., 2022). The FCPs cited lack of time to explore the barriers and obstacles to patients returning-to-work in depth, and lack of confidence in addressing some of the psychosocial issues underlying work absence.

Similar to our current findings, Byrne et al.’s (2014) analysis of GP consultations with patients with medically unexplained symptoms identified interactional ‘tussles’ and negotiation regarding work absence. However, in contrast to our findings, the tussles they observed related to GPs’ reluctance to administer ‘fit notes’ to patients who had requested to be signed-off from work, and they did not identify any discussion of occupational support or advice. These previous findings highlight the value of the use of VSWs trained in providing vocational support to people who have been signed-off from work, as we found that the VSWs had the time, confidence and skills to engage in detailed discussions with individuals about their work absence and plans to RTW.

Andersen et al. (2014) identified similar findings to ours regarding the value of therapeutic support in facilitating RTW discussions. In testing a RTW intervention delivered to individuals in Denmark, they found that participants highlighted factors such as the confidence and trust they had in the RTW professional, and the extent to which the professional understood their concerns, as being important in influencing their engagement in RTW discussions. However, Anderson et al stopped short of identifying specific strategies used by professionals to achieve this in consultations, as we have done in this article.

### Strengths and limitations

The depth of understanding that we were able to generate through the use of theme-oriented discourse analysis is a strength of this study. Presenting extended extracts from only four VSW-participant consultations (based on the analysis of a sample of eight) clearly limits the potential to generalise the findings to other VSW consultations in the WAVE feasibility study. However, the aim of this analysis was to understand the interactional and discursive strategies the VSWs and participants employed when discussing work issues and RTW, not to extrapolate these findings to all consultations.

The influence of the researchers’ role in the analysis must be acknowledged, giving due consideration to the influences of their professional backgrounds and subjective viewpoints. In particular, the fact that the researchers were closely involved in the development and testing of the vocational advice intervention, could have had the potential to influence the way the consultation data were interpreted. However, a reflexive approach was adopted throughout, in which the researchers attended to, and acknowledged their subjective engagement with the data during the analysis.

## Conclusion and implications

Consultations that focus on supporting people to return-to-work have been shown to be complex interactional settings, often involving back-and-forth negotiations of competing agendas. VSWs put in considerable interactional ‘work’ to encourage individuals to address obstacles to return-to-work and engage with return-to-work planning. In the process they utilised a range of strategies to provide therapeutic support and to attempt to reassure individuals about their return-to-work concerns.

These findings have implications for both research and clinical practice. The discursive patterns and interactional strategies identified will directly inform amendments to the training for a larger number of VSWs to deliver vocational support in the forthcoming WAVE pilot and main trial. This will test whether delivery of vocational support by VSWs leads to fewer days’ work absence over 6 months in people who receive a ‘fit note’ from their GP.

The findings also have broader implications for informing conversations that healthcare professionals have with their patients about work-related issues and concerns. ‘Patient activation’, in terms of empowering patients and building their confidence to be actively involved in decisions about their health, is a goal outlined in the UK NHS Long Term Plan (2019). The interactional strategies we identified can help healthcare professionals to negotiate patients’ concerns and worries about RTW. This can function to build individuals’ confidence to be actively engaged in discussions about returning-to-work, which can ultimately benefit their health and wellbeing.

## References

[bibr1-13634593221148446] Andersen MF Nielsen K Brinkmann S (2014) How do workers with common mental disorders experience a multidisciplinary return-to-work intervention? A qualitative study. Journal of Occupational Rehabilitation 24(4): 709–724. 24532340 10.1007/s10926-014-9498-5 PMC4229648

[bibr2-13634593221148446] Aylward SM (2016) Overcoming barriers to recovery and return to work: Towards behavioral and cultural change. In: Schultz I Gatchel R (eds) Handbook of Return to Work. Handbooks in Health, Work, and Disability, vol 1. Boston, MA: Springer, pp.119–139.

[bibr3-13634593221148446] Brown P Levinson SC (1987). Politeness: Some Universals in Language Usage. Cambridge: Cambridge University Press.

[bibr4-13634593221148446] Byrne P Ring A Salmon P , et al. (2014) Tussles and Rollovers: Negotiating sickness certification in primary care. Advances in Applied Sociology 4(12): 247–260. Available at: http://www.fitforworkeurope.eu/ (accessed 9 March 2022).

[bibr5-13634593221148446] Fit for Work Europe. (2020). Available at: http://www.fitforworkeurope.eu/ (accessed 9 January 2023).

[bibr6-13634593221148446] Goffman E (1955) On face-work: An analysis of ritual elements in social interaction. Psychiatry 18(3): 213–231. 13254953 10.1080/00332747.1955.11023008

[bibr7-13634593221148446] Kendall NAS Burton AK Main CJ , et al., on behalf of the Flags Think-Tank (2009) Tackling Musculoskeletal Problems: A Guide for the Clinic and Workplace—Identifying Obstacles Using the Psychosocial Flags Framework. London: The Stationary Office.

[bibr8-13634593221148446] Letrilliart L Barrau A (2012) Difficulties with the sickness certification process in general practice and possible solutions: A systematic review. European Journal of General Practice 4: 219–28. Available at: https://www.longtermplan.nhs.uk/ (accessed 7 March 2022). 10.3109/13814788.2012.727795 23205966

[bibr9-13634593221148446] O’Brien K Cadbury N Rollnick S , et al. (2008) Sickness certification in the general practice consultation: The patients’ perspective, a qualitative study. Family Practice 25(1): 20–26. 18245795 10.1093/fampra/cmm076

[bibr10-13634593221148446] O’Keefe BJ Shepherd GJ (1987) The pursuit of multiple objectives in face-to-face persuasive interaction: Effects of construct differentiation on message organization. Communication Monographs 54: 396–419.

[bibr11-13634593221148446] Roberts C Sarangi S (2005) Theme-oriented discourse analysis of medical encounters. Medical Education 39(6): 632–640. 15910440 10.1111/j.1365-2929.2005.02171.x

[bibr12-13634593221148446] Saunders B Foster NE Hill JC , et al. (2022) First contact practitioners’ (FCPs) and general practitioners’ perceptions towards FCPs delivering vocational advice to patients with musculoskeletal conditions: A qualitative investigation of the implementation potential of the I-SWAP initiative. Journal of Occupational Rehabilitation 32(1): 147–155. 34241768 10.1007/s10926-021-09992-5 PMC8858917

[bibr13-13634593221148446] Silva-Junior JSD Fischer FM (2014) Long-term sickness absence due to mental disorders is associated with individual features and psychosocial work conditions. PLOS ONE 9(12): e115885. 25531900 10.1371/journal.pone.0115885 PMC4274157

[bibr14-13634593221148446] Sowden G Main CJ van der Windt DA , et al. (2019) The development and content of the vocational advice intervention and training package for the study of work and pain (SWAP) trial (ISRCTN 52269669). Journal of Occupational Rehabilitation 29: 395–405. 29982957 10.1007/s10926-018-9799-1 PMC6531387

[bibr15-13634593221148446] Waddell G Burton AK (2006) Is Work Good for Your Health and Wellbeing? London: TSO.

[bibr16-13634593221148446] Wynne-Jones G Artus M Bishop A , et al. (2019) Effectiveness and costs of a vocational advice service to improve work outcomes in patients with musculoskeletal pain in primary care: A cluster randomised trial. Pain 159(1): 128–138. 10.1097/j.pain.0000000000001075 28976423

